# Development of a battery-free ultrasonically powered functional electrical stimulator for movement restoration after paralyzing spinal cord injury

**DOI:** 10.1186/s12984-019-0501-4

**Published:** 2019-03-08

**Authors:** Monzurul Alam, Shuai Li, Rakib Uddin Ahmed, Yat Man Yam, Suman Thakur, Xiao-Yun Wang, Dan Tang, Serena Ng, Yong-Ping Zheng

**Affiliations:** 1 Department of Biomedical Engineering, The Hong Kong Polytechnic University, Hung Hom, Kowloon, Hong Kong SAR, China; 2 Department of Chemical Sciences, Tezpur University, Tezpur, 784028 India; 3 Guangdong Work Injury Rehabilitation Center, Guangzhou, China; 4 Community Rehabilitation Service Support Centre, Hospital Authority, Hong Kong SAR, China

**Keywords:** Functional electrical stimulation, Wireless power, Ultrasound, Piezoelectric

## Abstract

**Background:**

Functional electrical stimulation (FES) is used to restore movements in paretic limbs after severe paralyses resulting from neurological injuries such as spinal cord injury (SCI). Most chronic FES systems utilize an implantable electrical stimulator to deliver a small electric current to the targeted muscle or nerve to stimulate muscle contractions. These implanted stimulators are generally bulky, mainly due to the size of the batteries. Furthermore, these battery-powered stimulators are required to be explanted every few years for battery replacement which may result in surgical failures or infections. Hence, a wireless power transfer technique is desirable to power these implantable stimulators.

**Methods:**

Conventional wireless power transduction faces significant challenges for safe and efficient energy transfer through the skin and deep into the body. Inductive and electromagnetic power transduction is generally used for very short distances and may also interfere with other medical measurements such as X-ray and MRI. To address these issues, we have developed a wireless, ultrasonically powered, implantable piezoelectric stimulator. The stimulator is encapsulated with biocompatible materials.

**Results:**

The stimulator is capable of harvesting a maximum of 5.95 mW electric power at an 8-mm depth under the skin from an ultrasound beam with about 380 mW/cm^2^ of acoustic intensity. The stimulator was implanted in several paraplegic rats with SCI. Our implanted stimulator successfully induced several hindlimb muscle contractions and restored leg movement.

**Conclusions:**

A battery-free miniature (10 mm diameter × 4 mm thickness) implantable stimulator, developed in the current study is capable of directly stimulating paretic muscles through external ultrasound signals. The required cost to develop the stimulator is relatively low as all the components are off the shelf.

**Electronic supplementary material:**

The online version of this article (10.1186/s12984-019-0501-4) contains supplementary material, which is available to authorized users.

## Background

According to the Christopher and Dana Reeve foundation survey (2013), there are roughly 5.4 million people currently suffering from Spinal Cord Injury (SCI) in the United States of America [7]. The World Health Organization report (2013) stated that, each year, 250,000 to 500,000 people newly suffer SCI worldwide [5]. There are significant challenges for treating patients with SCI as it has widespread consequences for many body functions which not only include limb paralysis but also bladder, bowel, respiratory and cardiovascular dysfunctions [24, 66].

There are few rehabilitation therapies available for SCI patients. But, unfortunately, patients with severe SCI do not benefit from these conventional physical rehabilitation therapies [22]. For these severely paralyzed patents, Functional Electrical Stimulation (FES) is probably the only option for movement restoration [45]. FES is a method to activate the healthy neuromuscular tissues below the injury site, and thus produce muscle contractions to provide functions to the paretic limbs [59]. The main principle of FES is the triggering of muscle contractions by electrically activating intramuscular nerve branches [31]. FES can be utilized to bypass an injured neural tissue to activate healthy tissue [3, 39]. FES is commonly used during aerobic exercise training for individuals with SCI to benefit their functional rehabilitation [15]. FES can also act as an adjunctive to traditional physical therapy to improve ambulatory function [8]. Furthermore, applying FES to SCI paraplegics along with a custom-made stimulation schedule can increase the muscle mass of the paretic body parts, and thus improve the sitting pressure distribution and reduce spasticity [14]. Repetitive use of FES can also promote motor learning and recovery of function by regaining previously learned motor skills which have been lost due to the local damage of neural circuits [26]. Besides, FES can keep SCI patients physically active [13], and thus improve cardiorespiratory fitness [32], enhance exercise performance [38] and increase muscle strength [19]. Hence, FES is widely used in SCI rehabilitation due to its functional and therapeutic benefits.

However, there are several limitations existing in the current implanted FES systems. The first and foremost limitation is powering [57]. As most of the implantable stimulators require batteries as their power source, this significantly increases the size of the implant. A battery occupies more than 80% of the total size of an implantable device [12]. Apart from the size, battery-powered stimulators require a second surgical procedure to replace the battery every few years [65]. This surgery is often unsuccessful, resulting in a complete loss of the implant or even its functions. To combat these issues, several hybrid systems were developed that utilize a rechargeable battery with radio frequency (RF) charging capabilities [42, 51]. These systems have been widely used for FES applications [52, 67]. However, despite several attractive features, the implant does not completely eliminate the need for a battery and thus still poses the risk of secondary surgery to replace the module as battery life is projected to be 5–10 years, depending on its discharge and recharge cycles [36, 41].

The objective of this study was to develop a wirelessly powered miniature neuromuscular stimulator that can be implanted deep inside the human body for FES-induced movement restorations after paralysis. In the present work, preference was given to acoustic power transfer over other power transduction modalities such as electrical and electromagnetic transfer, based on the efficiency, energy absorption by the skin and depth of transmission into the body. A number of recent studies have shown the efficacy of successful power transmission to biomedical implants via ultrasound energy [21]. To develop an ultrasonically powered FES system, we first tested a number of piezoelectric materials and selected the best material with the highest output power. Next, we determined the best conditioning circuit to convert piezoelectric signals into stimulation pulses. After that, we prototyped the stimulator, and coated it with biocompatible materials. The size of the implant has been greatly reduced as the battery is absent from the device. Finally, we implanted the stimulator in paralyzed rats and tested the stimulation-induced muscle contraction and leg movements in the anaesthetized condition.

## Methods

The methods of the current study are broadly divided into three parts: searching for suitable piezoelectric materials for the development of an implantable wireless stimulator, prototyping the stimulator with an optimum conditioning circuit, and in vivo testing of the stimulator in paralyzed animals. Several piezoelectric materials were tested in this study for maximum power transduction through skin. An optimum voltage conditioning circuit was then designed and tested for conversion of the piezoelectric voltage into stimulation pulses. The prototype stimulator was then coated with biocompatible materials and implanted into spinal-injured rats for movement restoration via muscle stimulation. The following sections describe these methods in detail.

### Selection of piezoelectric material

**Fig. 1:**
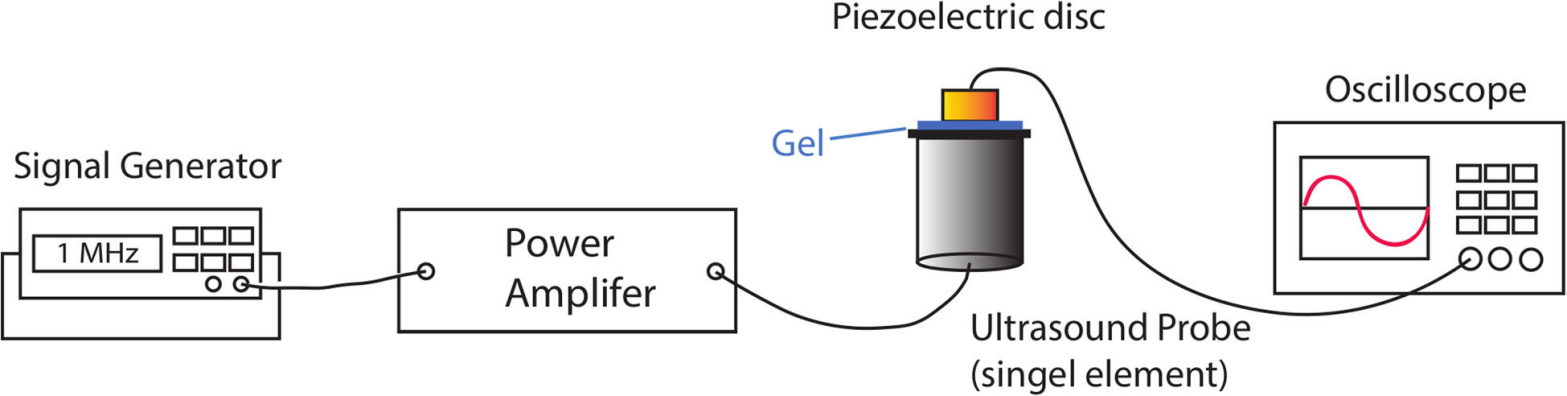
Benchtop setup for determination of the best piezoelectric material for prototyping the stimulator. Ultrasound signal was generated by the ultrasound probe driven by a power amplifier with input signal from a function generator. Ultrasound gel was used between the ultrasound probe and the piezoelectric disc. The piezoelectric voltage was measured across a load resistance connected to the piezoelectric disc

**Fig. 2:**
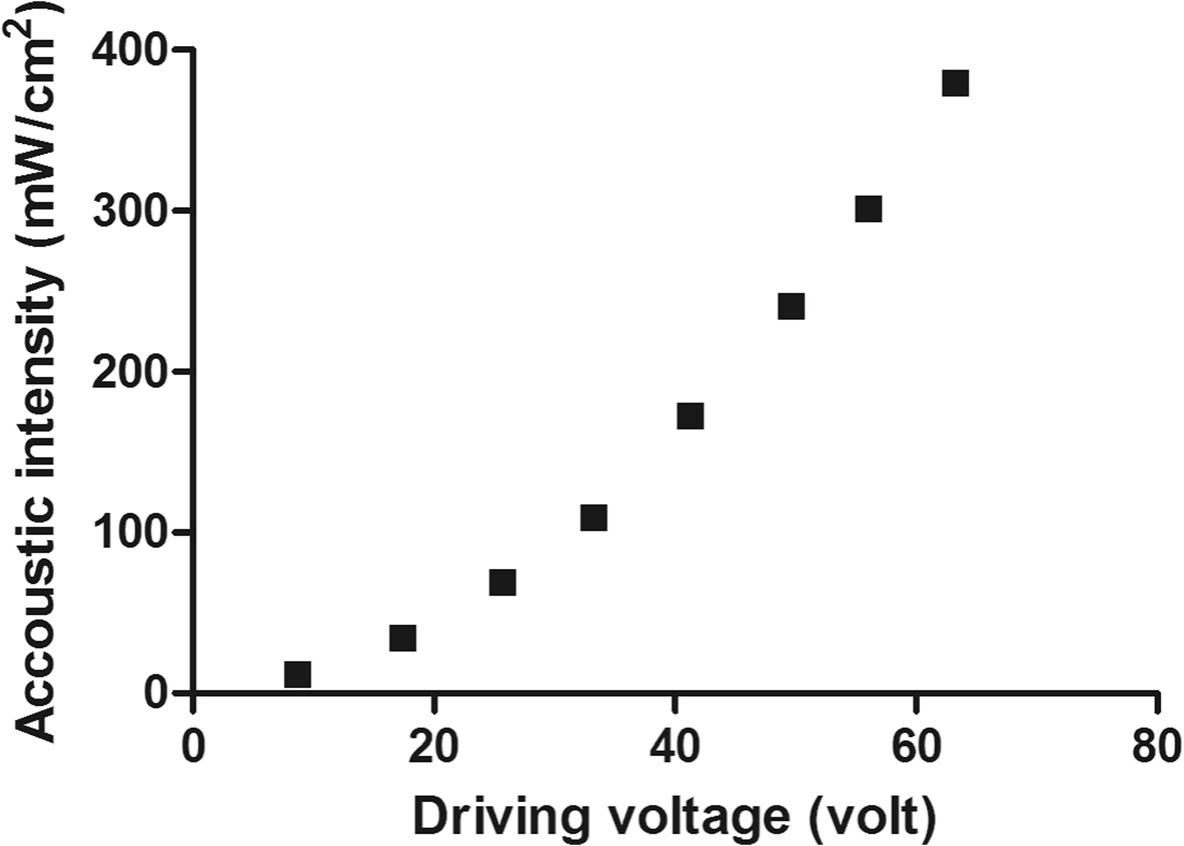
Spatial peak temporal average (SPTA) intensity of the ultrasound signals at 8 mm in water. The ultrasound signals were generated at different driving voltages by an external ultrasound probe

In the search for the best piezoelectric material for prototyping of our stimulator, a total of three lead zirconate titanates (PZT4, PZT5, PZT8) and one single crystal ceramic (BaTiO_3_) were primarily identified based on their piezoelectric coefficients and market availability. All the piezoelectric discs used in this study were 10 mm in diameter with an optimum frequency of approximately 1 MHz. Three piezoelectric discs of each material were tested in a benchtop setup to find the best material as illustrated in Fig. 1. A signal generator (AFG3021, Tekronix, United States) was used to generate a 1 MHz sine wave signal, and a 5 watt power amplifier (Dahan Radio Studio, China) was used with a plane ultrasonic probe (4902, DOBO, China) to produce ultrasound irradiation. The acoustic intensity values of the ultrasound irradiation for different input signal intensities are presented in Fig. 2. The detailed methods for measuring the ultrasound intensity using a hydrophone are described elsewhere [40].

The materials were tested by placing the piezoelectric disc directly on top of the ultrasonic probe with ultrasonic gel as medium. By recording which disc could generate the highest piezoelectric voltage at the same input signal, the disc with the highest efficiency was determined. Transduced piezoelectric voltage was measured using an oscilloscope (HDO9000, Teledyne LeCroy, United States) across a load resistance connected to the piezoelectric disc. The effect of ultrasound intensity on generating the piezoelectric voltage was first determined by changing the input signal amplitude. The effect of changing the load resistance (1 kΩ & 10 kΩ) was also investigated; thus, driving current capabilities were also examined using this setup. A total of four different settings were tested in this experiment.

### Conditioning circuit design

Since the piezoelectric disc generates an approximately 1 MHz signal, a fast acting diode is required to rectify the signal. Selection of the rectifier diode was mainly based on three parameters: forward bias voltage, reverse breakdown voltage and maximum operating frequency. The forward bias voltage was preferred to be as low as possible (< 300 mV) for minimal loss, the reverse breakdown voltage should be > 5 V and the maximum operating frequency was required to be at least 1 MHz for our application. Based on these parameters, a RF Schottky Barrier Diode (1SS351, On Semiconductor, United States) was selected.

**Fig. 3:**
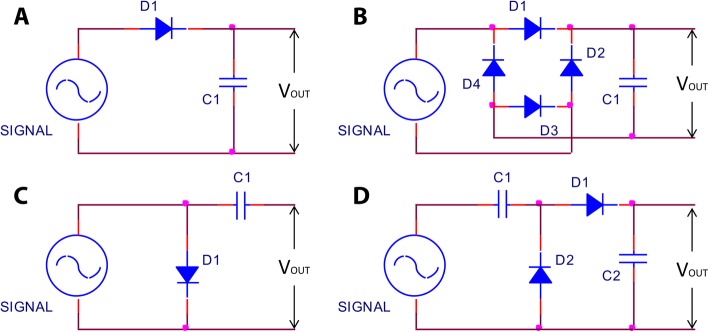
Schematic diagram of four tested conditioning circuits, **a** Half-wave rectifier and filter; **b** Full-wave rectifier and filter, **c** Circuit used by Larson and Towe [37]; and **d** Villard voltage doubler circuit

Four different conditioning circuits (Fig. 3) were tested to rectify a 1 V (p-p), 1 MHz sine wave signal produced by a function generator (AFG3021, Tekronix, United States). The conditioning circuits were a half-wave rectifier and filter; full-wave rectifier and filter; Villard voltage doubler; and the configuration used by Larson and Towe [37]. By comparing the generated output voltage across a load resistance (10 kΩ) of each conditioning circuit, the best conditioning circuit was determined. The input cycles were kept constant at 500 cycles during this test.

After determining the best conditioning circuit, the selected circuit was further tested from 20 to 500 cycles of sine wave (1 MHz) at a constant input voltage of 5 V (p-p) in order to determine the required minimum input signal cycles to generate maximum output voltage. In addition, to test the effects of input voltage amplitude on the generated stimulation voltage, the input voltage was increased from 1 V (p-p) to 10 V (p-p) with a fixed 500 cycles of sine wave for the same conditioning circuit.

**Fig. 4:**
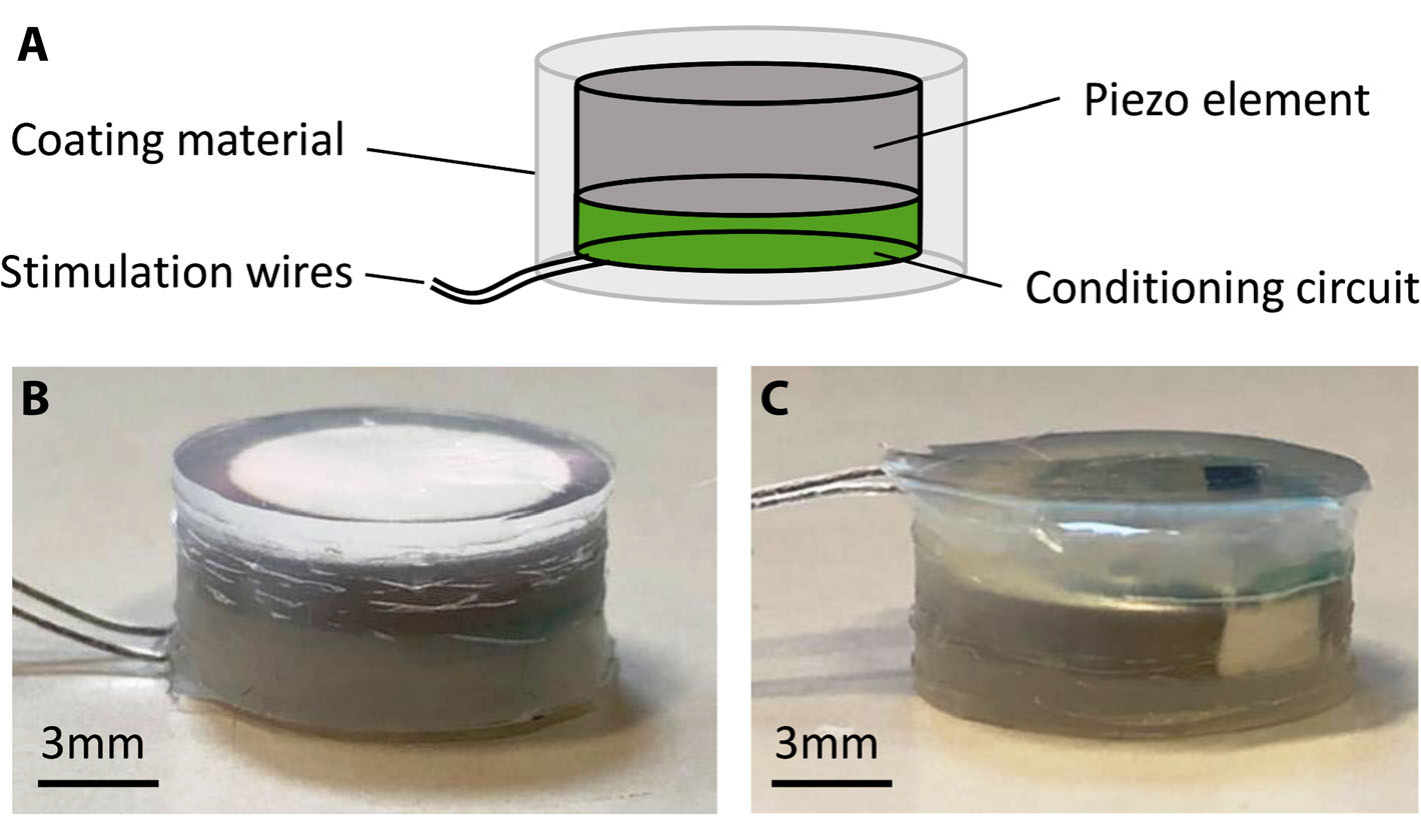
**a** Layout of our piezoelectric stimulator (PolyUStimulator). The conditioning circuit and the connections of the stimulation wires are on one layer of the double-layered PCB, while the other layer is connected to a piezoelectric disc. Finally, the stimulator is encapsulated with a biocompatible silicone elastomer. **b**-**c** Face-up and face-down side views of the prototype stimulator

After testing the best conditioning circuit to convert a 1 MHz sinusoidal signal into a stimulation pulse, a small (diameter: 8 mm, thickness: 0.6 mm) double-layer printed circuit board (PCB) was designed and developed. The diodes and capacitors were soldered on one side of the PCB while the other side was soldered to the piezoelectric disc. A pair of Teflon-coated stimulation wires (AS-632, Cooner Wire, Chatsworth, CA, United States) was also connected to the PCB for outputting the stimulation pulses. The entire package was then encapsulated with biocompatible coating (Fig. 4).

### Biocompatible encapsulation

As it was aimed to be implanted into the body, the prototype stimulator was encapsulated (Fig. 4) with biomaterials for electrical insulation and biocompatibility. The electronics of the stimulator were coated with a synthesized polymer (polyurethane) by the dip coating method. The polyurethane was first dissolved in solvent (N,N-Dimethylacetamide) and then coated as a thin layer on the stimulator. After drying at 75 °C for 12 h in a forced convection oven (OF-02G, JEIO TECH Co. Ltd., South Korea), another layer of FDA-approved biocompatible silicone elastomer (Shantou Chaonan Xiancheng Hengchang Silicone Material Factory, China) was applied on the stimulator. For ease of implantation, 1-mm thick silicone coating was made to fully embed the stimulator inside the silicone. Before curing, all the stimulators were sterilized, placed into the coating material, and then placed at rest under room temperature (25 °C) to ensure that all the bubbles had been removed, as the formation of bubbles will lower the ultrasound propagation and thus lower the stimulation voltage. The stimulators were cured at 45 °C for 3 h using the same oven.

The effects of coating hardness and the curing time on the stimulator’s output voltage were also determined in a prior experiment. To determine the effects of hardness on the acoustic impedance (and thus the generated output voltage), two levels of silicone, level 30 and level 60 (the higher the number, the harder the coating) were tested for a constant curing time (3 h). Furthermore, to determine the effects of curing time on the acoustic impedance, two curing times (1 h vs. 3 h) were also tested for both hardness levels.

### Measuring the stimulation signal from attenuated ultrasound

**Fig. 5:**
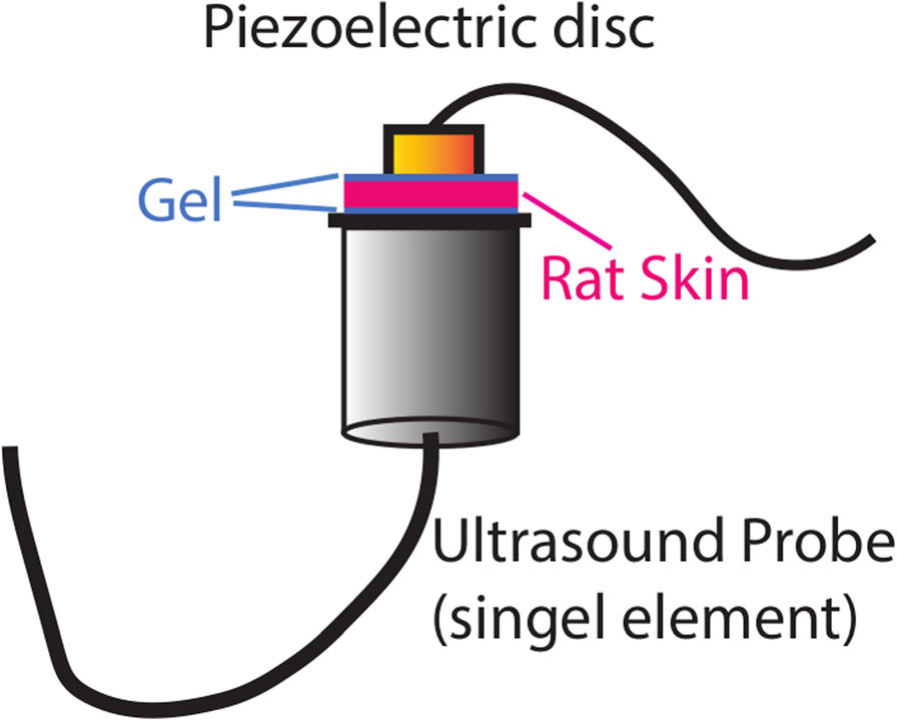
Setup for measuring the stimulator’s output voltage from attenuated ultrasound signal through rat skin

Acoustic energy encounters a significant attenuation while passing though biological tissue [18]. Hence, rat skin was used in between the ultrasound probe and the stimulator (Fig. 5) to test if our prototyped stimulation can generate a sufficient stimulation voltage from the attenuated ultrasound signal. The effect of external ultrasound intensities through the skin to the generated output voltage of the stimulator was then measured across different loads (1 kΩ and 10 kΩ). The ultrasound cycles were kept constant at 500 cycles during this experiment.

### Animal surgery

To test the functionality of the stimulator, in vivo tests were conducted in three adult sprague dawley rats (~ 245 g). The surgical procedures were performed in accordance with the guidelines and approval of the Animal Subjects Ethics Sub-committee of The Hong Kong Polytechnic University.

The rats were first anaesthetized with isoflurane gas (1.5 to 2.5%) administered via a facemask and maintained at the optimum level throughout the surgery. To prevent hypothermia, body temperature was maintained at 37 °C using a heating pad (H-KWDY-III, Quanshi Experimental instrument, China). The surgical site (head, thoracic region and left hindlimb) were carefully shaved with a clipper, and disinfected by povidone-iodine (Betadine®, Mundipharma, Switzerland) followed by 70% ethanol. The eyes were covered by sterile ophthalmic gel (Lacryvisc®, Alcon, France) to prevent dryness. Surgical tools were sterilized by autoclave and UV-ray prior to surgery. To carry out a spinal hemisection at T7 spinal cord level, a laminectomy was performed to expose the spinal segments. The dura was removed from the midline to facilitate the hemisection. Sharp microscissors were then used to cut the spinal cord from the midline to the lateral left. First, one blade tip of the scissors was passed through the left side of the spinal cord. After the insertion, the other blade of the scissors was closed and the left dorsal and ventral columns of the spinal cord were cut from the midline. To confirm the hemisection, the scissor tips were passed through the portion of the spinal cord to cut any remaining tissue. Then, a sterile cotton gauge was placed in the space if there was any bleeding. The muscles were then sutured using 4.0 Vicryl (ETHICON®, NJ, USA).

A head-plug with three different channels (2 stimulation channels, and 1 piezoelectric channel) was made before the implantation surgery. During the surgery, two small skin incisions were made on the head and the back of the rat. A small pocket was made near the lumbar region of the rat and the stimulator connected to the head plug was inserted through the incision of the head using forceps and placed into the pocket. To fix the stimulator, the wires from the stimulator were sutured to the paravertebral muscle. Four screws were used to anchor the head plug to the rat’s head and dental cement was used for stabilization. To stimulate the hindlimb muscles, multi-stranded Teflon-coated stainless-steel wires (AS632, Conner wire, CA, USA) were inserted into both the hindlimb gastrocnemius (GS) and extensor digitorum longus (EDL) muscles by a method described previously [4]. In brief, a small notch was made at the tip of the wires to create stimulation electrodes and the wires were then inserted into the left hindlimb muscles and anchored with sutures (Additional file 1: Figure S1). The skin was then sutured, and the stimulation was tested acutely via an external voltage stimulator (DS2A, Digitimer, UK).

The rat was allowed to recover for a week and its urinary bladder was manually expressed (3 times/day). Oral antibiotics (Enrofloxacin 0.6 ml/ 100 ml water) and analgesics (Buprenorphine HCL 0.5 mg/kg, S.C.) were administered twice daily for 3 days. Fresh fruit and juice was provided in the cage for first recovery.

### Stimulation experiment

**Fig. 6:**
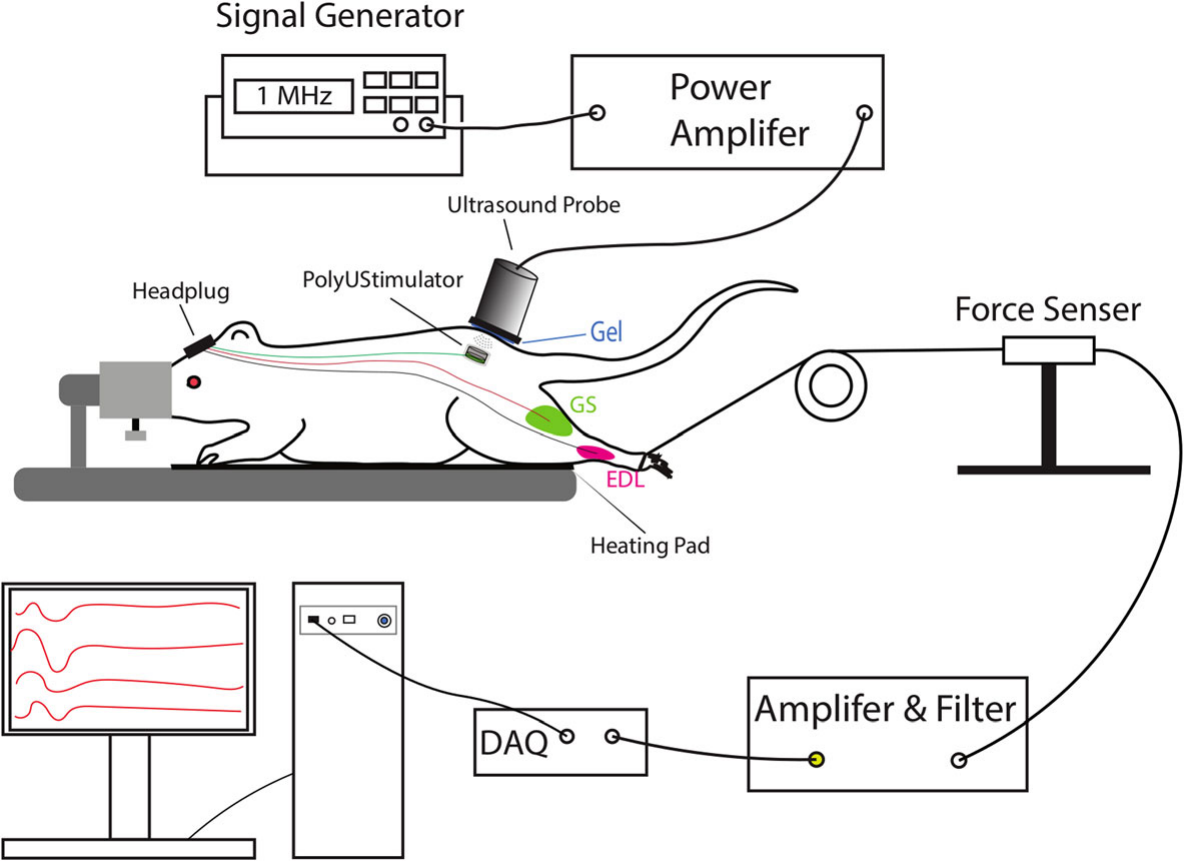
Experimental setup for in vivo stimulation. A 1 MHz sinusoidal signal was amplified to drive the external ultrasound probe. The ultrasound signal captured by the implanted piezoelectric stimulator to stimulate hindleg muscles. Force exerted by the muscle contraction was captured by a custom force sensor and then amplified and digitized for display on a computer screen

For FES testing, rats were anaesthetized with isoflurane gas (1.5 to 2.5%) administered via a facemask throughout the experiment. A stimulation test was performed by applying ultrasound irradiation to the implanted piezoelectric stimulator after applying ultrasound gel to the skin. The stimulation voltage was generated by the implanted stimulator. These voltages were used to stimulate the gastrocnemius (GS) and extensor digitorum longus (EDL) muscles one after another. The stimulation thresholds for these muscles were first determined by using a conventional isolated voltage stimulator (DS2A, Digitimer, USA). For successful muscle contraction, a 50 ms burst of stimulation (10 pulses at 200 Hz) was delivered at 3 s intervals. The movements were captured by placing an accelerometer (MPU6050, Arduino, Italy) on the toe end of the foot. To test the force generated by the muscle contractions from the stimulation burst, a force measurement tool was used. A thread from the force sensor tied to the rat’s ankle via a pulley was utilized to sense the force as a function of voltage (Fig. 6). Prior to the in vivo experiments, calibration was carried out with the use of 50 g, 100 g and 150 g deadweights. Ultrasound parameters were changed to generate different muscle contractions. The measured force was digitized (Power1401-3A, Cambridge Electronics Design Ltd., United Kingdom) and stored in a computer for further analysis. Adequate rests were given between the testing sections to avoid muscle fatigue from the FES.

**Fig. 7:**
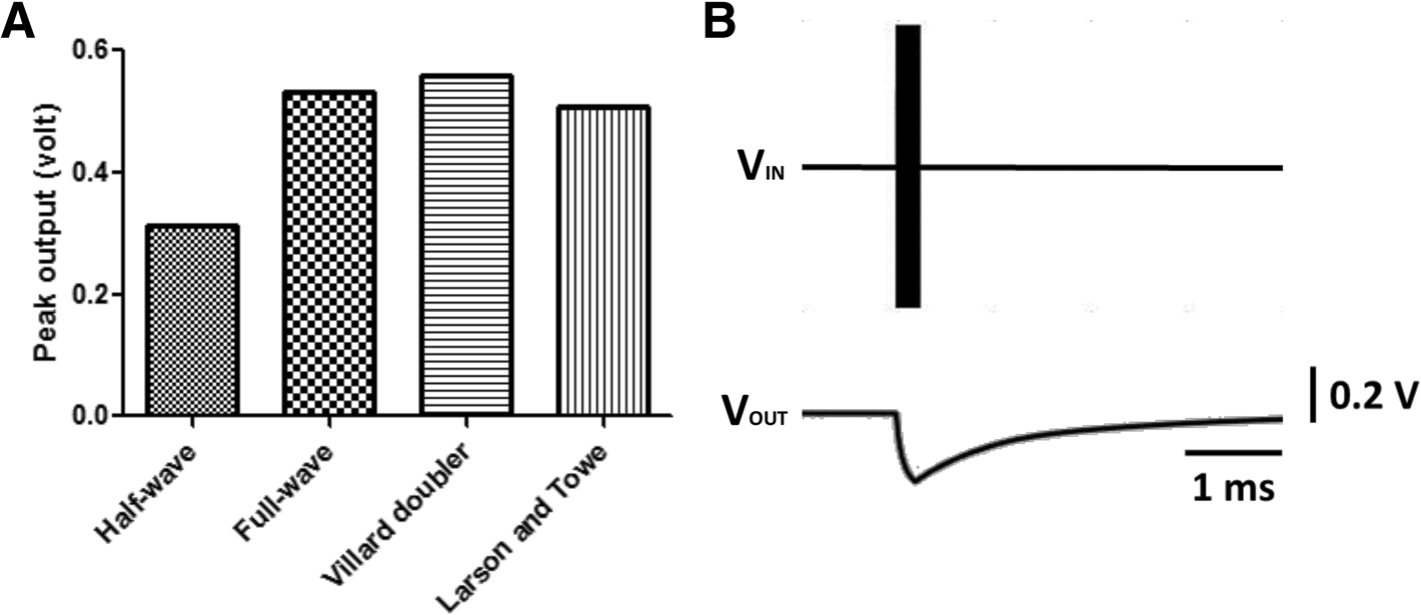
**a** Comparison of peak output voltage of different conditioning circuits for 1 V (p-p), 1 MHz sinusoidal input signal. **b** Output pulse generated by the Villard voltage doubler circuit from a 200 μs sinusoidal burst at 1 MHz. The pulse shape is similar to conventional FDA-approved electrical stimulators

### Data analysis and statistics

All the data were converted into a suitable format using a custom script written in MATLAB (MathWorks Inc., Natick, MA, USA). All statistical analyses were performed using GraphPad Prism (GraphPad Software Inc., LaJolla, CA, USA). For the analysis of piezoelectric voltage generated by four different piezoelectric materials, one-way ANOVA was used to compare the output voltage of each disc to determine the significance of difference. The effects of curing time and coating hardness were analyzed by first normalizing the output voltage (generated output voltage divided by the reference voltage of the disc). After calculating the normalized voltage of each disc, the mean was taken on discs with the same coating condition. Then an unpaired t-test was used to compare the effects of curing time and coating hardness. All data are reported as mean (± standard error).

## Results

### Best piezoelectric material for implantable stimulator

The initial focus of our study was to find the best piezoelectric material in the market that can generate the highest stimulation voltage from 1 MHz ultrasonic irradiation inside the body. The experimental results of testing four different piezoelectric materials (PZT4, PZT5, PZT8 and BaTiO_3_) under four different experimental settings are shown in Table 1. The average output voltage of three discs from each material was calculated. The maximum average voltage for each experimental setting is highlighted in blue (Table 1), suggesting the best material for that particular setting. It was found that PZT8 performed the best and PZT4 performed the worst among the four piezoelectric materials tested in all of the experimental settings. The average voltage generated by PZT8 was significantly higher (*p* < 0.05; two-way ANOVA; Bonforroni post hoc test) than the average voltage generated by PZT4 discs. No significant difference was found in the generated voltages of discs made of PZT5 and BaTiO_3_.

**Table 1:** Experimental results of the output generated voltages from the piezoelectric discs made from different piezoelectric materials

Material	PZT4 (Volt)	PZT5 (Volt)	PZT8 (Volt)	BaTiO_3_ (Volt)
Experimental setting				
Ultrasound = 1 MHz, 109.59 mW/cm^2^. Load = 1 kΩ	10.70 ± 0.63	11.27 ± 0.50	14.17 ± 1.88	11.53 ± 0.61
Ultrasound = 1 MHz, 109.59 mW/cm^2^. Load = 10 kΩ	10.53 ± 0.95	13.07 ± 1.47	15.73 ± 2.64	12.73 ± 1.33
Ultrasound = 1 MHz, 379.92 mW/cm^2^. Load = 1 kΩ	18.47 ± 1.22	19.07 ± 0.42	24.73 ± 3.51	20.07 ± 0.64
Ultrasounds = 1 MHz, 379.92 mW/cm^2^. Load = 10 kΩ	18.40 ± 1.59	22.20 ± 2.75	27.73 ± 3.88	21.40 ± 1.11

### Optimum voltage conditioning

FES utilizes mono- or bi-phasic rectangular pulses for nerve or muscle stimulation [44]. Negative (cathodic) pulses have been found to be more effective than positive (anodic) pulses for successful stimulation [53]. To find an optimum conditioning circuit to generate maximum voltage and standard shape stimulation pulses from the piezoelectric signal, four different conditioning circuits (see Fig. 4) were tested in this study. It was found that among all the four conditioning circuits, the Villard voltage doubler circuit generates the highest amount of stimulation voltage for a 1 V (p-p), 1 MHz sinusoidal signal (Fig. 7a). The full-wave rectifier and filter circuit, however, can generate similar voltage to that generated by the Villard doubler, but requires more components. The waveform generated by the circuit reported by Larson and Towe [37] was different to conventional pulse stimulation (data not shown), while our Villard voltage doubler circuit with a 10 kΩ load produced pulse-shaped stimulation from a 200 μs sinusoidal burst (1 MHz) as shown in Fig. 7b.

**Fig. 8:**
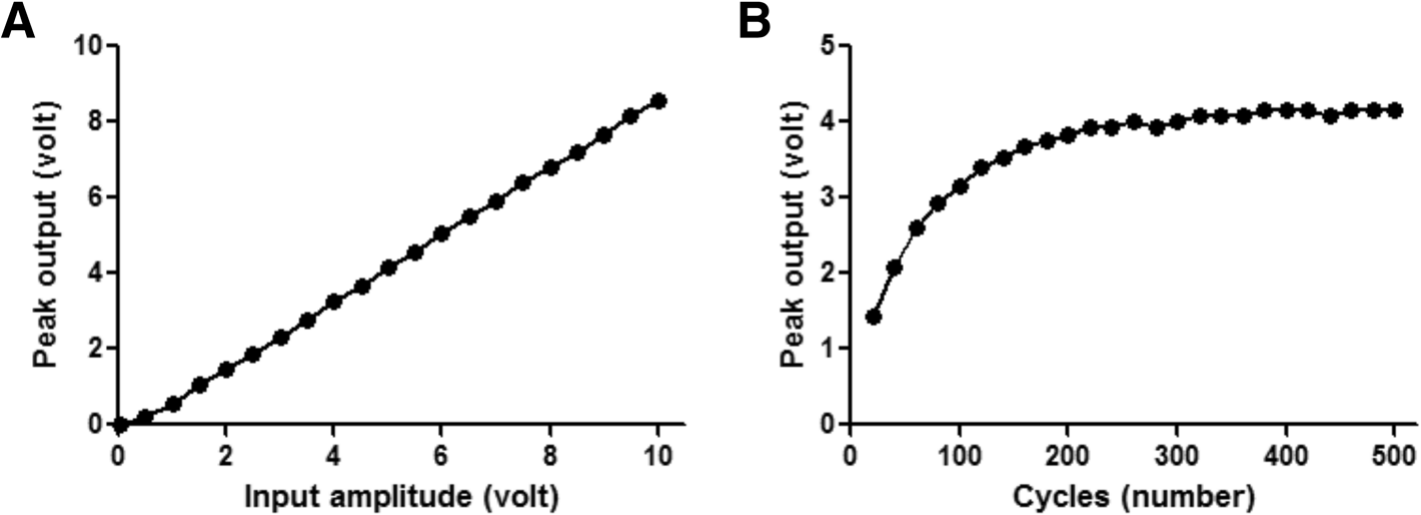
Effects of changing input signal. **a** Amplitude to the generated output voltage at a constant 500 cycles. **b** Cycles to the generated output voltage at a constant 5 V (p-p) input signal at 1 MHz and 10 kΩ load

### Effects of input signal parameters on the conditioned output voltage

After finalizing the Villard voltage doubler as the conditioning circuit for our stimulator, the effects of changing the input signal amplitude and cycles on the output voltage were determined. Figure 8a shows the effect of changing the input signal amplitude on the generated output voltage of the conditioner circuit. The response appears to be quite linear. But the changes of signal cycles resulted in non-linear changes of output voltage of the conditioning circuit to the plateau value of around 240 cycles (Fig. 8b).

### Effect of curing time and coating hardness on the stimulator output voltage

**Fig. 9:**
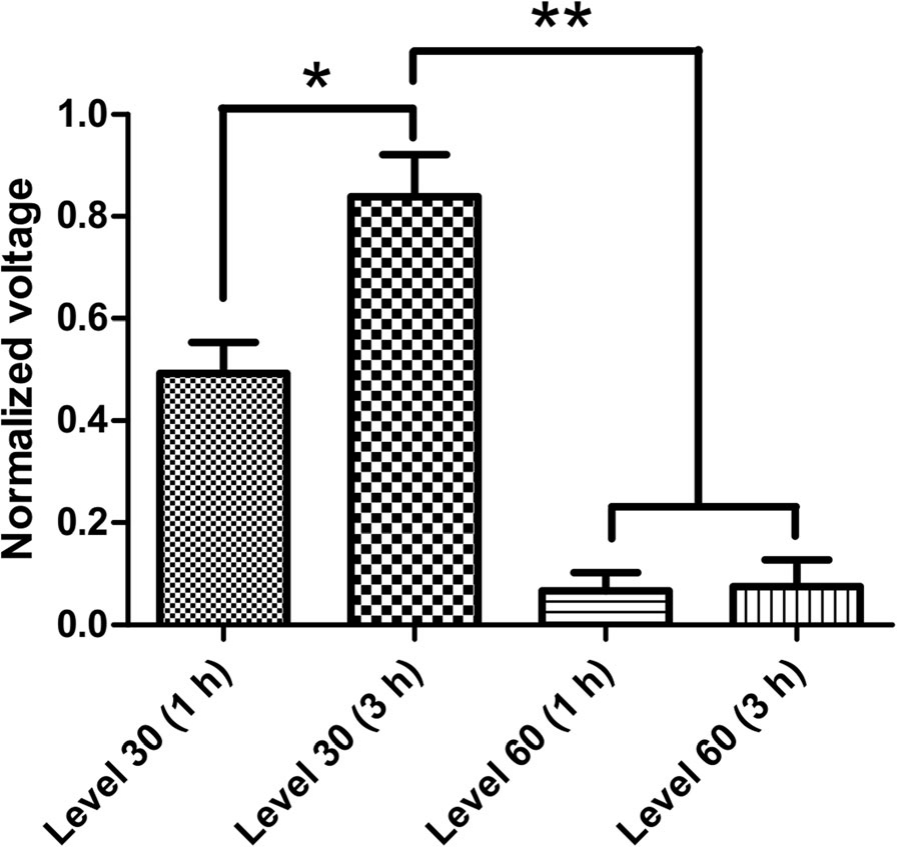
Effects of coating hardness and curing time on the piezoelectric voltage of PZT8 discs. * and ** indicate significant difference at *p* < 0.05 and *p* < 0.001

All the three PZT8 discs could reach around 27.73 V (p-p) without coating (see Table 1). To test the effects of coating hardness and curing time on acoustic loss (and thus the generated output voltage by selected piezoelectric discs), two levels of silicone, level 30 and level 60, were tested with two different curing times (1 h and 3 h). Based on the normalized output voltage, it was found that the harder the material, the lower the generated voltage, and the longer the curing time, the higher the generated output voltage (Fig. 9). Based on this study, we encapsulated our stimulator with silicone with a hardness level of 30 under 3 h of curing.

### Energy transduction efficiency of our piezoelectric stimulator

**Fig. 10:**
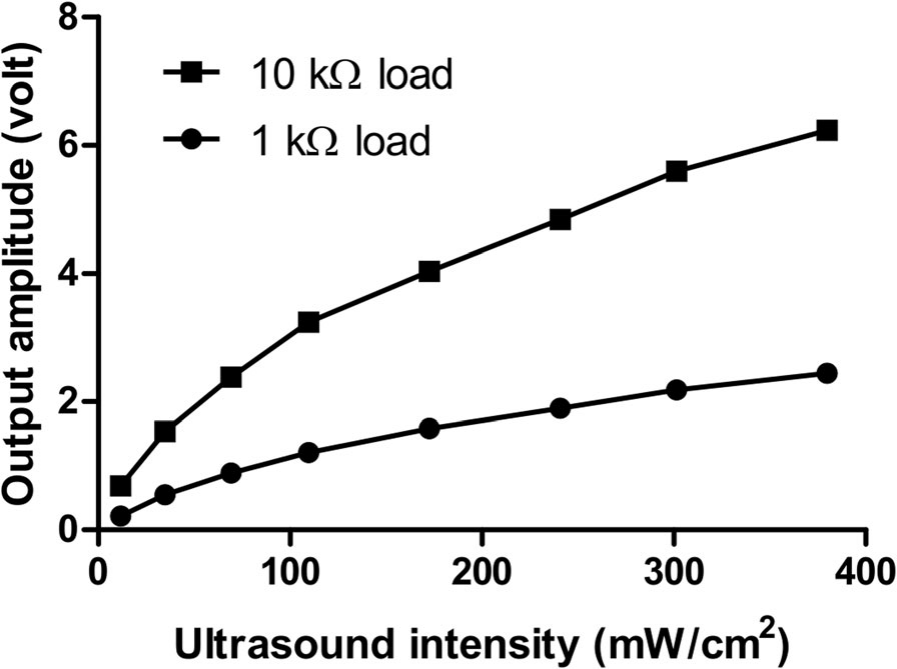
Stimulator output voltage to attenuated ultrasound signals through the skin under low (10 kΩ) and high (1 kΩ) load resistances

To test if our prototyped stimulation can generate sufficient stimulation voltage from attenuated ultrasound, ex vivo rat skin was used in between the ultrasound probe and the stimulator, and the stimulator’s generated output voltages for both low (10 kΩ) and high (1 kΩ) loads were measured for different ultrasound intensities (see Fig. 5). As expected, the stimulator output voltage increased with increasing ultrasound intensity (Fig. 10). It was found that the output voltage was much higher with the low (10 kΩ) than with the high (1 kΩ) load; however, the generated power depends on both voltage and current outputs. The stimulator was found capable of harvesting 5.95 mW of electric power at an 8-mm depth under the skin from an external acoustic intensity (SPTA) of only 379.92 mW/cm^2^.

### Evoked movements by piezoelectric stimulation in vivo

**Fig. 11:**
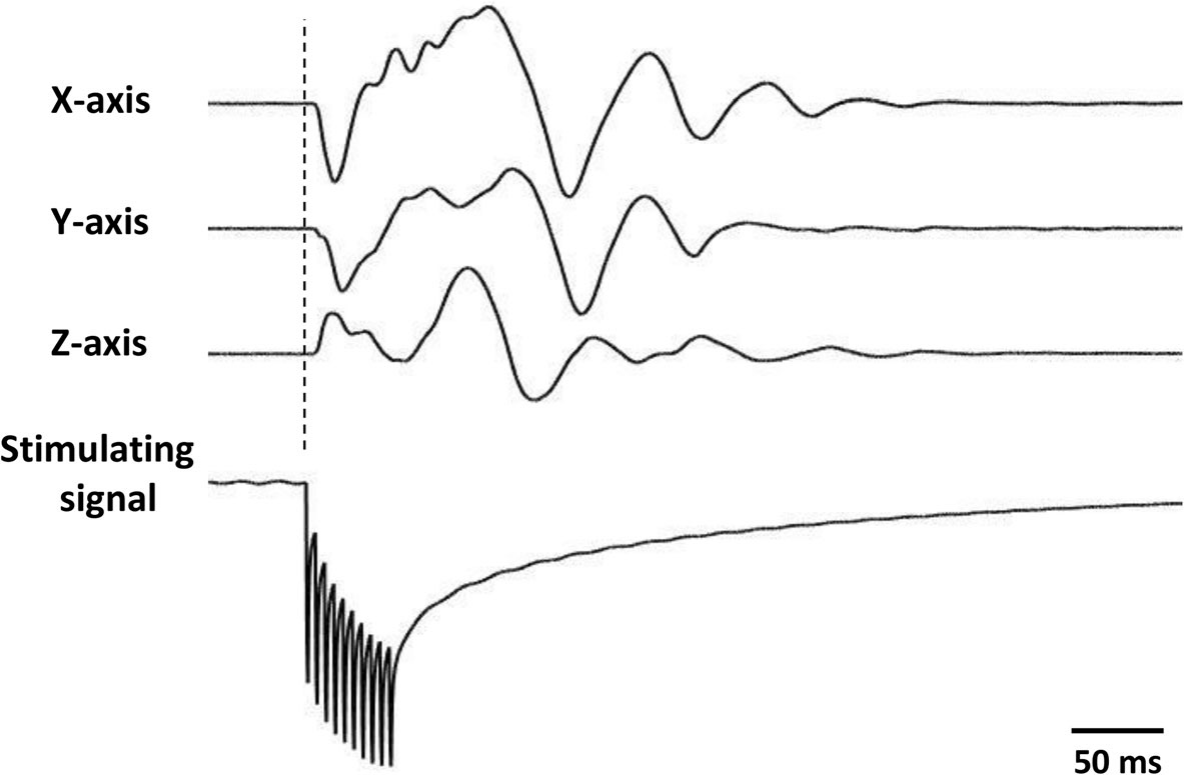
3-axis accelerometer data (first three rows) during piezoelectric stimulation of the extensor digitorum longus (EDL) muscle with 50 ms bursts (ultrasound parameters: 1 MHz, 200 cycle, 200 Hz reputation rate) with our PolyUStimulator. The bottom row shows the piezoelectric stimulation pulses for the implanted stimulator


Three SCI rats with brown sequard syndrome (spinal cord hemisection) were implanted with our prototype stimulator. The stimulation electrodes were implanted into left hindleg muscles (GS: gastrocnemius and EDL: extensor digitorum longus) to test the efficacy of our piezoelectric stimulation to induce muscle contraction and leg movement. An ultrasound probe was placed on top of the skin targeting the implanted stimulator (as shown in Fig. 6) to send ultrasound signals to the disc to generate piezoelectric voltage. The generated stimulation voltage was collected through the wire connected to the head plug and fed back to either channel (GS or EDL) for muscle stimulation. The stimulation induced left hindleg movement as shown in Additional file 2: Video S1. An accelerometer was connected to the left hindleg to capture the leg movement. Figure 11 shows the 3-axis accelerometer data during muscle stimulation from the implanted stimulator. Prior to the piezoelectric voltage stimulation, we tested both muscle (GS and EDL) thresholds from standard voltage stimulation. The stimulation thresholds were found to be consistently different for all the animals tested (GS: > 6 V; EDL: 0.3–0.9 V). Due to the lower threshold, we only stimulated EDL muscle using our implanted stimulator.

**Additional file 2: Video S1.** A representative rat implanted with PolyUStimulator demonstrating hindleg movements in response to external ultrasound. The light indicates the onset of ultrasound burst. (MOV 1656 kb)


**Fig. 12:**
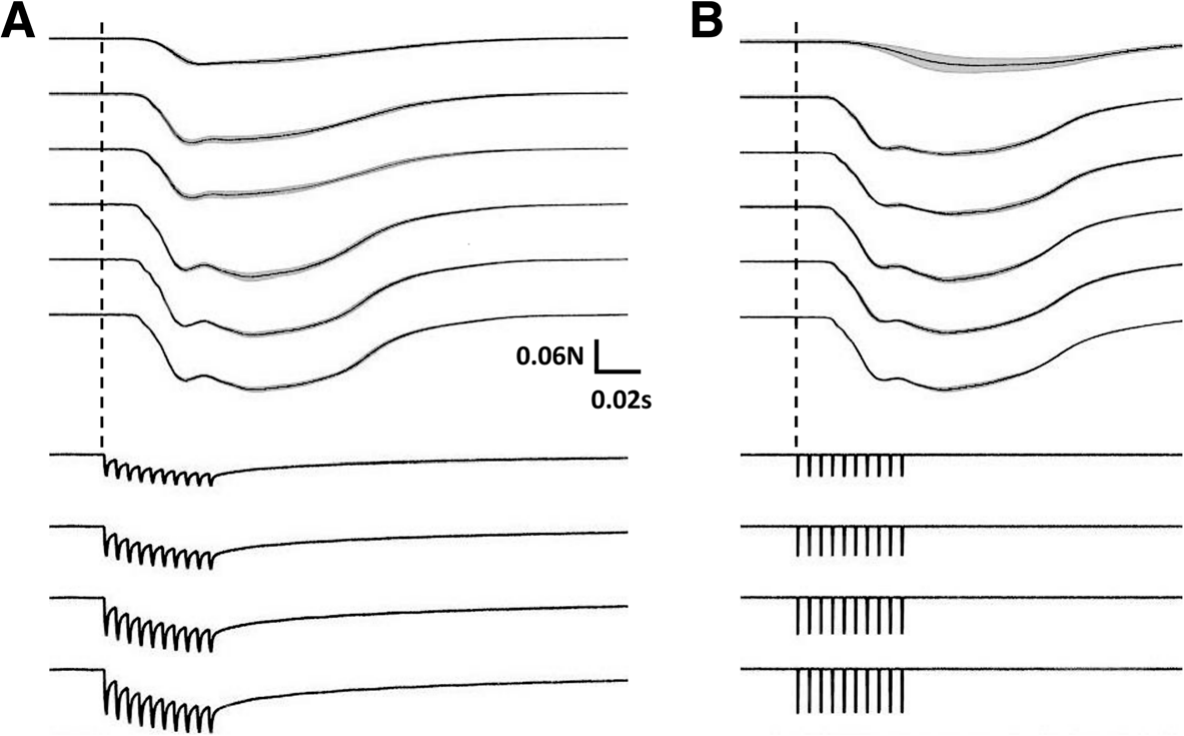
Different force exerted from different stimulating signals **a** PolyUStimulator and **b** conventional isolated voltage stimulator. In each figure, force changes are displayed in the upper section and colour-matched stimulating signals are shown in the bottom section

**Fig. 13:**
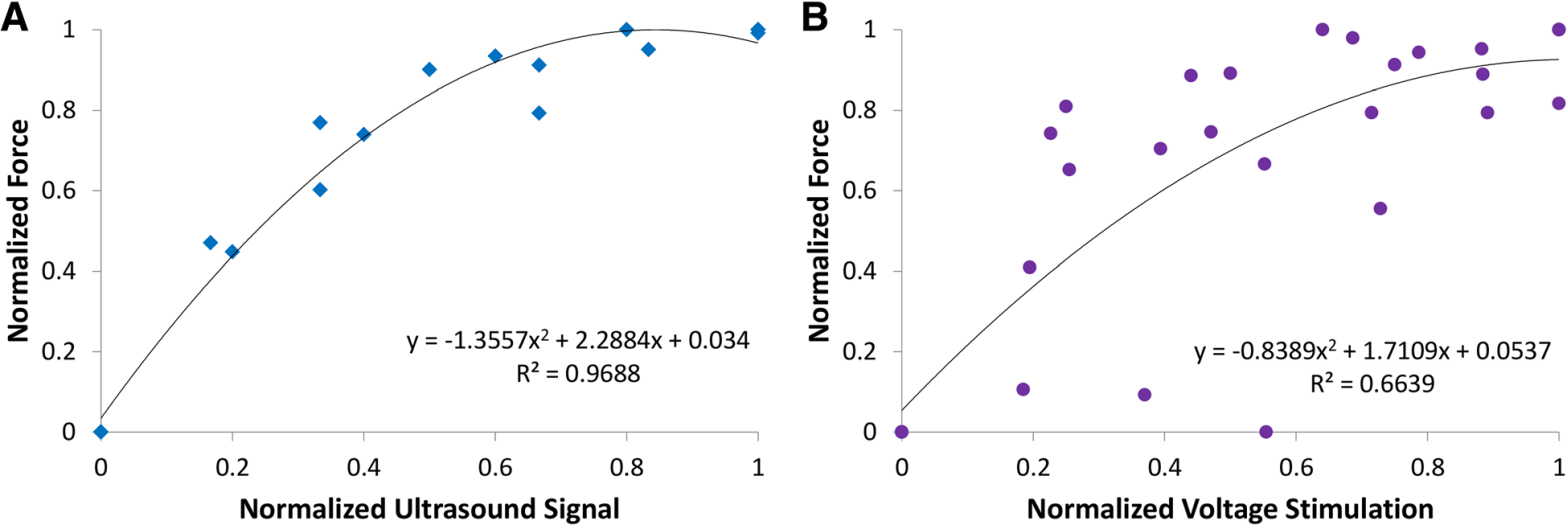
Comparisons between normalized forces generated by **a** Ultrasound-meditated piezoelectric and **b** electrical voltage stimulations in anaesthetized rats (*n* = 3). Piezoelectric stimulation was delivered by our PolyUStimulator, whereas pure electrical stimulation was delivered by a conventional isolated stimulator (DS2A, Digitimer, USA)

To test the force exerted by the muscle contractions from the piezoelectric stimulation burst, a thread from the force sensor was tied to the rat’s ankle via a pulley (see Fig. 6). The isometric force generated during different stimulations of the left EDL muscle is shown in Fig. 12. The piezoelectric stimulation delivered by our PolyUStimulator generated similar forces as compared to the conventional isolated voltage stimulator (DS2A, Digitimer, USA). Figure 13 shows the group data (*n* = 3) of two different stimulation modalities. Ultrasound that delivered piezoelectric stimulation from our stimulator (PolyUStimulator) appears to have better muscle recruitments (*R*^*2*^ = 0.9688 vs. *R*^*2*^ = 0.6639) compared to the conventional FES system.

## Discussion

Electroceuticals, i.e., treating disease or anomalies with electrical impulses, is immerging and may be the future of modern medicine [55]. But delivering power to these electrical implants, deep inside the body, remains a critical challenge [1]. To address this, different wireless powering modalities have been investigated. Wireless powering of implantable devices utilizes an energy transduction method to generate electrical energy from vibrational, electromagnetic, electrostatic, infrared radiant and/or ultrasound energy, through specific conversion [23]. Recent developments of implantable medical devices suggest that it might be more feasible to utilize wireless power transfer for electrical stimulation therapy compared to the classical power supply methods, such as battery implants [20]. The method of using harvested energy from external sources to stimulate nerve or muscle would be more endurable and could help to avoid multiple surgeries for replacing the battery or wire as the power can be wirelessly delivered to the implant [47]. There are several energy transduction methods which can possibly be implemented to power implantable devices such as optical [49], thermal gradient [68], vibrational energy [50], mid to far field radio energy [30], inductive power transfer [16], near-field capacitive coupling [34], and acoustic/ultrasound power transfer [11]. Table 2 summarizes recent developments of implantable medical devices based on these technologies. Our developed PolyUStimulator is one of the devices among them.

**Table 2:** Comparison of several implantable devices employing different energy transduction technologies

Publication	Method	Frequency (MHz)	Depth (mm)	Medium	Transmitter area (cm^2^)	Receiver area (cm^2^)	Input Power^a^ (mW)	Output Power^a^ (mW)	Efficiency (%)
[62]	IC	2400	15	Porcine tissue		0.78	0.15	0.0023	1.53
[27]	MF	1600	> 50	Tissue volume	< 36	0.031	500	0.2	0.04
[48]	IC	1500	< 30	Air	< 346	0.06	3200	15.7	0.49
[29]	FF	1000	1500	Air		20.4	15,000	32	0.21
[2]	MF	1600	42	Porcine tissue	< 36	0.53	800	0.45	0.056
[63]	NCC	0.25	1	Human skin	< 6.25	< 6.25		< 100	
[61]	NCC	4	0.2	Insulation film	2	192			0.027
[33]	NCC	98	25	Monkey Skin	32	4	316		56
[56]	IC + APT	2 and 0.2	70	Water	0.78	0.78	6000	0.029	< 0.001
[46]	APT	1	105	Phantom	21.3	0.3	1750	28	1.6
[17]	APT	1	30	Mineral oil	0.07	0.014		0.1	
[54]	APT	1	5	Porcine tissue	4.91	4.91	2000	440	22
[35]	APT	1.85	21.5	Rodent tissue		0.006		≥0.35	
This Work	APT	1	8	Rat skin	7.07	0.78	1000	5.95	0.60

*IC* inductive coupling, *MF* mid-field, *FF* far-field, *NCC* near-field capacitive coupling, *APT* acoustic power transfer

^a^electrical

One of the most promising energy transduction methods is piezoelectric energy harvesting via ultrasonic signals as it has numerous advantages over inductive, electrostatic and mid-range RF power transmission such as lower tissue absorption, smaller transducers due to shorter ultrasound wavelengths, and higher power intensity threshold for safe operation [11]. In a recent study [60], a microelectromechanical system (MEMS) based broadband piezoelectric ultrasonic energy harvester (PUEH) was developed to overcome the general limitation of the piezoelectric ultrasonic energy transduction method, which includes a bulk piezoelectric transducer, power output fluctuations due to standing wave, biocompatible coating with better acoustic impedance, etc. This technology can be applied to nerve stimulation when precise alignment is maintained between transmitting and receiving elements [37].

The miniature (10 mm diameter × 4 mm thickness) implantable FES device proposed in this paper involves the use of direct stimulation converted from ultrasound signals without using a battery. Our stimulator differs from other recently developed wireless neurostimulators mainly in two aspects: i) design and ii) application. Our stimulator utilizes commercial off-the-shelf (COTS) components while most other wireless stimulators use custom integrated chips for energy harvesting. Hence, our stimulator is low cost, and easier to manufacture, assemble and troubleshoot. Regarding the application, our stimulator demonstrates more power-hungry FES application, while most wireless stimulators are used for neural stimulation of the peripheral nerve which requires relatively low energy compared to direct muscle stimulation [64].

### Properties of piezoelectric materials

Our results in Table 1 suggest that the generated voltages follow the order below:

$$ \mathrm{PZT}8>\mathrm{PZT}5>{\mathrm{BaTiO}}_3>\mathrm{PZT}4 $$

Previous studies on piezoelectric energy transduction efficiency indicate that the coupling coefficient, dielectric loss and mechanical quality factor are the dominant parameters that affect energy transduction [25, 58, 69]. The specifications of our tested materials are listed in Table 3.

**Table 3:** Specifications of four piezoelectric materials affecting energy transduction efficiency

Material	Coupling coefficient	Dielectric loss	Mechanical quality factor
PZT4	0.55	0.005	800
PZT5	0.74	0.02	70
PZT8	0.63	0.003	1100
BaTiO_3_	0.43	0.005	1300

Based on these three parameters, the selection of piezoelectric material should consider the properties of better coupling coefficient, lower dielectric loss and a higher mechanical quality factor. However, it is difficult to find the best material from Table 3. Furthermore, resonance frequency might have affected the output voltage as we used fixed 1 MHz ultrasound signals. But each piezoelectric disc may have an individual resonance frequency close to 1 MHz. As only three discs were tested in each group, it is hard to compare between different materials. However, among all of the tested materials, PZT8 quite clearly stood out as the best.

### Input-output relationship

The current study utilized the best piezoelectric material (PZT8) to maximize the generated output voltage with minimum ultrasound input. Ultrasound needed to travel across a 5–7 mm thickness of skin to reach the targeted PZT disc. It was observed that the output voltage increased quite linearly with the increase of input ultrasound intensity (Fig. 10). Ultrasound intensity or acoustic pressure can be expressed by the following equation [46]:

$$ A(x)=A0{\exp}^{-\alpha \left(\mathrm{f}\right)\mathrm{x}} $$

where A(x) = intensity, A0 = original intensity, α(f) = attenuation coefficient, and x = distance along the acoustic axis; α(f) and x in both setups were kept constant throughout the testing. Therefore, when the input intensity increased, the output voltage also increased. Apart from that, the voltage generated without skin was much higher than that with skin. This is simply because of the attenuation: α(f) of ultrasonic gel (without skin) is much lower than the α(f) of ham [9].

### Signal conditioning

The voltage doubler, full-wave rectifier filter and the circuit reported by Larson and Towe [37] generated almost the same amount of voltage for stimulation. However, the waveforms were quite different. The output voltage from the voltage doubler and full-wave rectifier filter was single pulse while that from Larson and Towe [37] was pulsating, hence very different from conventional electrical stimulation pulses used for FES. Furthermore, the circuit designed by Larson and Towe [37] short circuits the positive half of the piezoelectric sinusoidal signal, which will eventually cause heating of the surroundings. Heating affects the immune system, calcium metabolism and DNA synthesis thereby significantly affecting human health [43]. In contrast, the voltage doubler circuit produced nice monophasic stimulation pulses (Fig. 8). The full-wave rectifier filter circuit is also capable of generating similar monophasic pulses; however, it requires more electronic components than the voltage doubler circuit. Hence, the voltage doubler circuit was used in our stimulators.

### Ultrasound parameters to control stimulation pulses

Based on our results, increasing input cycles increased the output voltage up to 4.16 V (Fig. 8). The reason is that the 1 MHz input signal wavelength (1 μs) is too short to charge a capacitor in one cycle. As a result, increasing input cycles facilitate the storing process in the voltage pump capacitors and finally increase the output voltage. When the circuit is fully saturated, further increase of input cycle does not increase the output voltage. Another way to control the output voltage is to directly change the input signal amplitude (ultrasound intensity). An increased input intensity delivered a higher stimulation pulse. Hence, these two parameters were used to control the stimulation pulse’s amplitude and width.

### Biocompatible coating and matching layer

We used biocompatible materials to coat our stimulator before implantation into the animals. We know that PZT is toxic in nature. Hence, we first coated the PZT disc and the associated electronics with a biocompatible polymer. Then, we further coated the prototype with FDA-approved silicone elastomer. Two curing times and two hardness levels of the silicone were tested. The best combination was curing for 3 h using level 30 silicone (Fig. 9). It appears that the hardness of the coating material affects the acoustic impedance. According to [28], acoustic impedance equals the product of density of the material and acoustic velocity in the medium. The harder the material, the higher the density, which results in higher acoustic impedance. In the in vivo experiment, rat tissues were placed in between the ultrasonic probe and the silicone-coated PZT stimulator. For a coating with higher acoustic impedance, higher acoustic impedance mismatch between tissue and the coated interface may occur. As a result, most of the energy is reflected back and less ultrasound can reach the PZT disc [6]. To ensure higher efficiency in transmitting ultrasound across these media, suitable matching layers are needed. Since no matching layer was strategically designed and used in this study, a softer material (level 30 silicone) was preferred to minimize the acoustic impedance mismatch, and less reflection was present in the system, finally achieving a higher output voltage.

### In vivo functional stimulation by the implanted stimulator

In our in vivo experiment, we stimulated the left hindlimb muscles of the rat through intramuscular stimulation. Since the stimulation was delivered into the muscle, if we wanted to record the EMG from the muscle, it would saturate the EMG amplifier and no EMG signal would be observed due to high stimulation artifacts. Hence, instead of EMG, we used accelerometers to record the limb movements. Teflon-coated stimulation wires from our stimulator were passed into the hindlimb muscles via a head plug mounted on the rat’s head (shown in Fig. 6). The selection of appropriate muscle (gastrocnemius or extensor digitorum longus) for the stimulation was carried out by a jumper connected to the head plug. The stimulation pulses generated by our stimulator were delivered into the muscles via the coated wires and electrodes at the tips.

The results of the in vivo experiments confirm the possibility of applying ultrasound signal to energize a body implant that stimulates muscles to induce movements (Fig. 11). The isometric force produced by our stimulator is highly comparable with conventional isolated voltage stimulators (Fig. 12). Prolonged electrical stimulation leads to muscle fatigue; and during muscle fatigue, a force loss occurs as described previously [10]. However, it appears that the stimulation delivered by our piezoelectric stimulator may induce less fatigue than the conventional constant voltage stimulator as shown by better recruitments of muscle by the stimulation (Fig. 13). This may be due to the ramp-type monopolar stimulation rather than flat monopolar stimulation.

## Conclusions

The present study successfully demonstrated the feasibility of using external ultrasound signals to power an implanted piezoelectric stimulator to induce movements in spinal cord injured rats using functional electrical stimulation. The stimulator uses a PZT disc, a voltage doubler circuit and a pair of stimulating electrodes. Presently, this prototype is able to generate sufficient voltage to induce muscle contraction in anaesthetized rats. However, more piezoelectric materials should be tested in the future to discover even better ones with higher efficiency to generate a higher stimulation voltage with less transferred power. Apart from this, the parameter which affects the generated output voltage should first be determined in order to select the best material. In the current study, it appears that the dielectric loss and mechanical quality factor contributed a lot to the output voltage of the stimulator. Further study of the piezoelectric parameters may be needed to improve the design of our stimulator.

## Additional files

Additional file 1: **Figure S1.** in vivo placements of PolyUStimualtor and implantation of intramuscular stimulation electrodes into the left hindlimb muscles. Image is after 3 months of chronic implant (the rat was sacrificed with an overdose of anesthetics). (TIF 9487 kb)

## Abbreviations

BaTiO_3_: Barium titanate
FDA: Food and drug administration
FES: Functional electrical stimulation
PZT: Lead zirconate titanate
SCI: Spinal cord injury
