# Supplementary figures and images for: Development of a battery-free ultrasonically powered functional electrical stimulator for movement restoration after paralyzing spinal cord injury
Source: J Neuroeng Rehabil. 2019 Mar 8;16:36. doi: 10.1186/s12984-019-0501-4 (PMC6408863; doi:10.1186/s12984-019-0501-4)

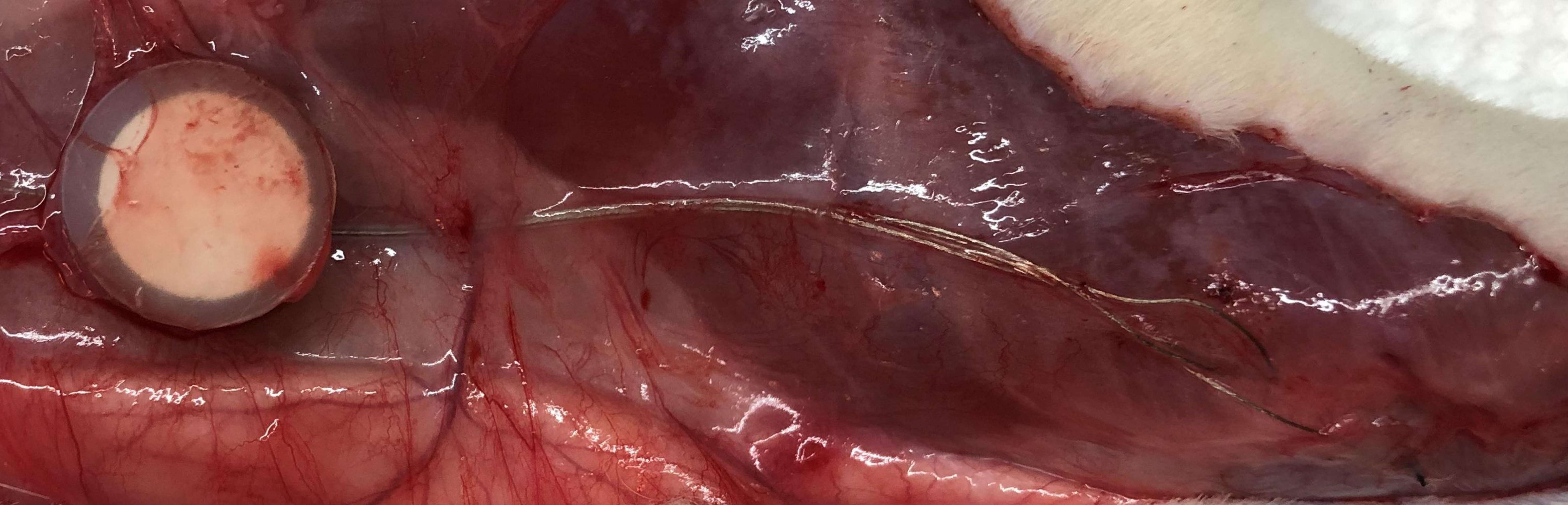

Supplement: Supplementary file 2 — Figure S1. in vivo placements of PolyUStimualtor and implantation of intramuscular stimulation electrodes into the left hindlimb muscles. Image is after 3 months of chronic implant (the rat was sacrificed with an overdose of anesthetics). (TIF 9487 kb) [file 12984_2019_501_MOESM1_ESM.tif]
